# Inhibition of O-GlcNAcylation Decreases the Cytotoxic Function of Natural Killer Cells

**DOI:** 10.3389/fimmu.2022.841299

**Published:** 2022-04-11

**Authors:** Daniel Feinberg, Parameswaran Ramakrishnan, Derek P. Wong, Abhishek Asthana, Reshmi Parameswaran

**Affiliations:** ^1^ Department of Pathology, Case Western Reserve University, Cleveland, OH, United States; ^2^ Department of Biochemistry, Case Western Reserve University, Cleveland, OH, United States; ^3^ The Case Comprehensive Cancer Center, Case Western Reserve University School of Medicine, Cleveland, OH, United States; ^4^ Division of Hematology/Oncology, Department of Medicine, Case Western Reserve University, Cleveland, OH, United States

**Keywords:** natural kiiler cells, O-GlcNAc, cytotocixicity, cytokines, post-translation modification

## Abstract

Natural killer (NK) cells mediate killing of malignant and virus-infected cells, a property that is explored as a cell therapy approach in the clinic. Various cell intrinsic and extrinsic factors affect NK cell cytotoxic function, and an improved understanding of the mechanism regulating NK cell function is necessary to accomplish better success with NK cell therapeutics. Here, we explored the role of O-GlcNAcylation, a previously unexplored molecular mechanism regulating NK cell function. O-GlcNAcylation is a post-translational modification mediated by O-GlcNAc transferase (OGT) that adds the monosaccharide N-acetylglucosamine to serine and threonine residues on intracellular proteins and O-GlcNAcase (OGA) that removes the sugar. We found that stimulation of NK cells with the cytokines interleukin-2 (IL-2) and IL-15 results in enhanced O-GlcNAcylation of several cellular proteins. Chemical inhibition of O-GlcNAcylation using OSMI-1 was associated with a decreased expression of NK cell receptors (NKG2D, NKG2A, NKp44), cytokines [tumor necrosis factor (TNF)-α, interferon (IFN-γ)], granulysin, soluble Fas ligand, perforin, and granzyme B in NK cells. Importantly, inhibition of O-GlcNAcylation inhibited NK cell cytotoxicity against cancer cells. However, increases in O-GlcNAcylation following OGA inhibition using an OGA inhibitor or shRNA-mediated suppression did not alter NK cell cytotoxicity. Finally, we found that NK cells pretreated with OSMI-1 to inhibit O-GlcNAcylation showed compromised cytotoxic activity against tumor cells *in vivo* in a lymphoma xenograft mouse model. Overall, this study provides the seminal insight into the role of O-GlcNAcylation in regulating NK cell cytotoxic function.

## Introduction

Natural killer (NK) cells are innate immune cells constituting 5%–20% of human peripheral blood mononuclear cells, and their main function is to kill cancer cells and virally infected cells (1). NK cell cytotoxic function is mainly regulated by inhibitory and activating cell surface receptors. Inhibitory receptors recognize major histocompatibility complex (MHC) class I molecules on normal cells, and this interaction results in signaling that inhibits cytotoxic function of NK cells. If target cells have downregulated MHC class I molecules, NK cells can recognize and kill those cells (2, 3). MHC class I is either absent or downregulated in cancer cells and virally infected cells. Cancer cells also express ligands for NK cell-activating receptors, and this interaction also leads to NK cell activation and its cytotoxicity against cancer cells (4). The signal from activating or inhibitory receptors in NK cells is responsible for shifting the balance toward either NK cell activation and elimination of target cells or NK cell inhibition, which prevents target cell killing. Another major factor regulating NK cytotoxicity is the induction of cytokine secretion after target cell recognition (5–7). These cytokines including interferon-γ (IFN-γ), tumor necrosis factor-α (TNF-α), and interleukin-10 (IL-10) as well as cytotoxic granules containing pore-forming perforin and apoptosis-inducing granzymes are key players of NK cell cytotoxicity (8–10).

**Figure 1:**
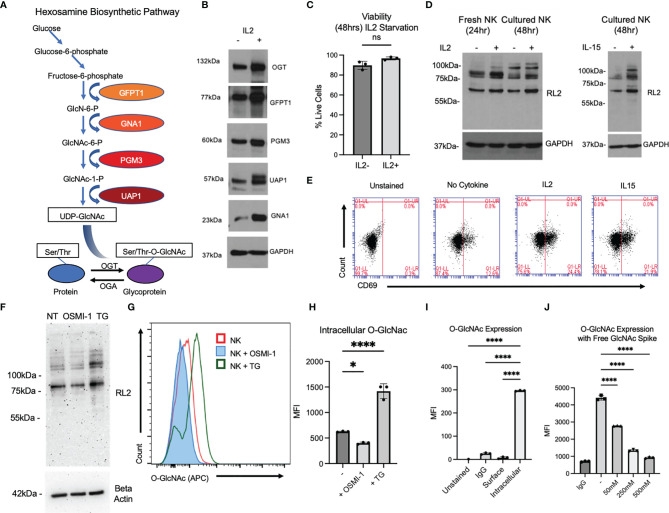
O-GlcNAcylation is present in primary NK cells and can be modified by activating cytokines and chemical inhibitors OSMI-1 (25 µM) and TG (50 µM). **(A)** Depiction of the hexosamine pathway, starting with glucose, which leads to UDP-GlcNAc formation for use by OGT. **(B)** Western blot in cultured primary NK cells of prominent enzymes in hexosamine pathway compared to loading control GAPDH with and without interleukin (IL)-2 (300 IU) for 24 h. **(C)** Countess viability data of trypan blue-stained primary NK cells after 48 h with or without IL-2. **(D)** Global O-GlcNAc protein expression by Western blot after stimulation with IL-2 in fresh and cultured primary NK cells. Cultured primary NK O-GlcNAc level was also measured by Western blot with and without IL-15 (10 ng/ml). **(E)** Flow cytometry analysis of CD69 expression in primary NK cells after 48 h of stimulation with activating cytokines. **(F)** Western blot of primary NK global O-GlcNAc levels with OSMI (25 µM) and TG (50 µM) for 36 h and beta-actin loading control. **(G)** Flow cytometry of intracellular O-GlcNAc levels in primary NK cells after OSMI and TG treatment for 36 h. **(H)** Graphical representation of values in panel **(G)** represented as median fluorescence intensity (MFI). **(I)** MFI of O-GlcNAc (RL2) flow antibody with primary NK intracellular content as positive control, showing no surface binding (on or off target) of flow antibody to antigen. **(J)** MFI of O-GlcNAc expression compared to matched immunoglobulin G (IgG) control with free GlcNAc as competition to antibody. (-) indicates positive control where no free GlcNAc was added. GlcNAc was spiked at increasing concentrations for 45 min during staining with (RL2) antibody at indicated concentrations. All MFIs are of the APC channel. NS indicates no significance. * indicates significance of p <.05, **** indicates p <.0001 as determined by one-way analysis of variance (ANOVA) with Dunnett’s multiple comparisons to untreated (-, IL2-, or intracellular) NK group mean or unpaired Student’s T-test depending on the number of samples being compared. Error bars are standard deviation of the mean. Panels **(C, F, G–I)** were performed n = 3. Panels **(B, D, E, J)** were performed n = 2. Individual data points shown are individual cellular cultures. Experiments shown are representative of 2 or 3 experiments performed as indicated above. All quantifications of Western blots in this figure can be found in **Supplementary Figure S1**.

For eliciting a productive immune response against cancer cells or viral infections, changes in metabolic pathways are also required in NK cells (11). NK cell activation resulting from interactions of activating receptors to its ligands is also dependent on metabolic pathways. Dysregulation of these metabolic pathways in NK cells often results in immune dysfunction and disease conditions. Cells depend on two major metabolic pathways: glycolysis and mitochondrial oxidative phosphorylation (12, 13). Metabolically active NK cells also depend on another energy source, glutamine, which is required for maintaining NK cell effector functions (14). Glucose and glutamine are also utilized in a metabolic pathway called hexosamine biosynthetic pathway (HBP) (15) that is responsible for the production of UDP-*N*-acetylglucosamine (UDP-GlcNAc), which is the substrate for a posttranslational modification, O-linked-β-N-acetylglucosamine glycosylation (O-GlcNAcylation) (**Figure 1A**).

*O*-GlcNAcylation occurs on intracellular proteins by addition of an *N*-acetylglucosamine (GlcNAc) from the precursor UDP-GlcNAc to serine and threonine residues on proteins (16). O-GlcNAcylation is carried out by two enzymes, O-GlcNAc transferase (OGT) that adds the monosaccharide, GlcNAc to the serine/threonine residue, and O-GlcNAcase (OGA) (16) that removes it (**Figure 1A**). The only enzyme capable of carrying out the O-GlcNAc addition is OGT from its UDP-GlcNAc source. This is in contrast to the over 500 kinases that have been discovered in the human genome, 188 of which were shown to be expressed in NK cells themselves (17). Given that UDP-GlcNAc is the only source/substrate of O-GlcNAc modifications, OGT/OGA have to be regulated in order to prevent minor metabolic perturbations from fundamentally altering global O-GlcNAcylation of the cell. While O-GlcNAcylation levels may be “buffered,” wider changes in the expression level of OGT and OGA outside of this tolerable range can have dramatic effects on immune cell function (18). Effects of O-GlcNAcylation changes in T cells are studied in more detail. When an inducible knockout of OGT was triggered in murine regulatory T-cells (Tregs), mice died of fatal autoimmunity, and when OGA was inhibited *via* Thiamet G (TG), there was an increase in Tregs (19). At the same time, there is evidence that O-GlcNAc levels also control Th17 and Th1 lineage differentiation through signal transducer and activator of transcription 3 (STAT3) and signal transducer and activator of transcription 4 (STAT4) (20). Most relevant to the findings in this article, Lund et al. (21) provide evidence that in effector T cells, global O-GlcNAcylation was increased along with OGT expression (without OGA changes) in response to activation and cytokine secretion. All of these data suggest that activation, differentiation, and function of T cells are regulated by O-GlcNAc levels. OGT is required for cell function, and a balance of O-GlcNAc levels is required for proper cell differentiation and cytotoxicity. T cells and NK cells are the predominant cytotoxic immune cells in humans. O-GlcNAcylation is known to play a role in activation and differentiation in T cells (22, 23), while its role in NK cell activation and cytotoxic function has not been explored yet. A previous study showed that O-GlcNAcylation levels in NK cells decreases in the presence of cancer cells (24); however, its role in NK cell effector function was not addressed. Here, we studied the role of O-GlcNAcylation in regulating NK cell cytotoxic function. We show that NK cell activation induced by IL-2 stimulation results in an increase in protein O-GlcNAcylation. Inhibition of O-GlcNAcylation using OSMI-1 (a chemical inhibitor of OGT) (25) treatment resulted in decreased expression of 1) NKG2D and NKG2A receptors; 2) cytokines including TNF-α and IFN-γ; 3) cytotoxic mediators perforin, granzyme B, soluble Fas Ligand, and granulysin resulting in reduced cytotoxic function of NK cells. In support of our *in vitro* data, mice injected with OSMI-1-pretreated NK cells showed decreased cytotoxic function, resulting in tumor progression, while control NK cells inhibited tumor growth. Thus, we provide *in vitro* and *in vivo* evidence that O-GlcNAcylation is essential for NK cell cytotoxic function.

## Materials and Methods

### Cell Lines and Culture

Jeko-1, Mino, MM.1S, and HEK293T cells were bought from the American Type Culture Collection (ATCC), and DAOY cells were a gift from Dr. Alex Huang (Case Western Reserve University, OH, USA). Jeko-1, Mino, MM.1S, and DAOY cells were cultured in RPMI-1640 (Sigma-Aldrich) supplemented with 10% fetal bovine serum (FBS) (Sigma-Aldrich) and 1% Pen/Strep (Hyclone). HEK293T cells were cultured in Dulbecco's Modified Eagle Medium (DMEM) (Sigma-Aldrich) with the same 10% FBS and 1% Pen/Strep supplementation. NK92 cells were a kind gift from Dr. Daniel Popkin (Innova Dermatology, TN, USA). They were cultured in Alpha-MEM media (Sigma-Aldrich) and supplemented with 12.5% FBS, 12.5% horse serum (Sigma-Aldrich), 0.02 mM folic acid (Acros), 0.1 mM 2-mercaptoethanol (Gibco), 1.5 g/L sodium bicarbonate (Fisher Scientific), 2 mM L-glutamine (Gibco), and 0.2 mM inositol (Acros). IL-2 (200 IU) was added fresh ever 2–3 days with media changes for NK92 cells. All cells were maintained at 37°C with 5% CO_2_ in the cell incubator. Glucose concentrations of media used for cell culture: 2 g/L for primary NK cells, as they were in RPMI-1640 media; 1g/L for NK92 cells, as they were cultured in Alpha-MEM; and 4.5 g/L for HEK293T cells, as we use DMEM-High Glucose. All media are from Sigma-Aldrich as mentioned above.

### Primary Natural Killer Cell Isolation and Expansion

Healthy donors were recruited *via* the Hematopoietic Stem Cell Core Facility at Case Western Reserve University, and 40 ml of blood in EDTA was collected per donor. Blood was isolated *via* Ficoll (GE Healthcare) gradient centrifugation to isolate peripheral blood mononuclear cells (PBMCs). Here, 1 × 10^7^ PBMCs were plated with supplemented RPMI media, IL-2 at 100 U/ml, and K562-irradiated feeder cells. IL-2 was replaced every 3 days, and feeder cells were replaced every 7 days. Experiments were run with cells ranging from Day 10 to Day 21. For certain experiments, IL2 dosing was increased up to 300IU as indicated in respective figure legends.

### Primary Natural Killer Cell Utilization *In Vivo*

Phosphate-buffered saline (PBS), untreated NK cells, or OSMI-1 (25 μM)-treated primary NK cells were utilized. Cells were incubated with or without OSMI-1 for 36 h. Primary NK cells were utilized from one of 2 donors on given injection days. Cells were only injected if they were between Day 9 and Day 17 post-isolation from the donor. Cells were washed 2x with PBS at 900 revolutions per minute (rpm) to remove OSMI-1 from media and injected as a cell suspension in PBS. Dilution required for 3 × 10^6^ cells was determined *via* Countess (Invitrogen) hemocytometer, and 100 µl of suspension was injected per mouse.

### Mice

Mice were bred at the Case Western Reserve University in accordance with the guidelines of the Institutional Animal Care Use Committee. NOD SCID IL-2r Gamma (NSG) mice were purchased from Case Western Reserve University Athymic Animal Core. Experiments were conducted using age- and gender-matched mice in accordance with approved institutional protocols. Mice were kept in their permanent housing at the imaging core facility for a minimum of 1 week before any experiments were performed.

### Tumor Mouse Models and Bioluminescent Imaging

Mice were anesthetized *via* isoflurane chamber with subsequent transfer to nose cone. Here, 1 × 10^7^ Jeko-luciferase-tagged cells were injected subcutaneously at the dorsal right flank. On days of imaging, mice were injected intraperitoneally (IP) with 150 mg/kg D-luciferin (PerkinElmer). After 8 days, initial tumors were measured via spectrum IVIS imaging system (PerkinElmer). Subsequent measurements occurred every 3-4 days. As indicated, primary NK cells isolated from one of two donors were injected at 3 × 10^6^ NK cells with 50 ng/mouse of human rhIL-15 (BioLegend). Luminescent images were utilized from 10 min post-injection of D-luciferin, and images were analyzed by Living Image software (PerkinElmer).

### Transfection/Transduction

pLKO and pLKO.1 shRNA plasmids targeting human OGA (TCRN0000133855; Mission Sigma) or human OGT (shOGT1-TRCN0000286200, shOGT2-TRCN0000286199, and shOGT3-TRCN0000293652; Mission Sigma) or green fluorescent protein (GFP) (Plasmid SHC005; Sigma) were cotransfected with lentivirus-packaging plasmids psPAX2 (Plasmid 12260; Addgene) and pMD2.G (Plasmid 12259; Addgene) at a ratio of 4:1:3 using X-treme GENE HP DNA transfection reagent (Sigma). HEK293T cells used X-treme Gene Transfection Reagent per protocol. Viral supernatants were collected at 48 and 72 h posttransfection. For viral transduction, 1 × 10^6^ cells were resuspended in 1 ml virus-containing medium with 10 µg/ml of polybrene into 1 well of a 12-well plate and centrifuged at 3,480 rpm for 90 min in a centrifuge that was prewarmed at 32°C. Here, 1 ml of fresh supplemented Alpha-MEM media were added to the cells per well, and cells are resuspended into a tissue culture flask of appropriate volume. The following morning, the cell culture medium is changed. Forty-eight hours later, selection with 2 μg/ml puromycin was started for 6 days. After 6 days, selection pressure was removed and cells were cultured per cell line and culture section. For HEK293T cell experiment determining shRNA plasmid efficacy: HEK293Ts were transfected at above ratio, the medium was changed the following morning, and O-GlcNAc levels were determined by flow cytometry 36 h posttransfection.

### *In Vitro* Cytotoxicity and Cytokine Analysis

Cancer cells were stained with efluor 670 according to protocol. NK92 or primary NK cells were incubated for 24 h without treatment or with OSMI-1/TG. The cells were then spun down at 900rpm for 5 min, and 160 μl of supernatant from treated groups was added to wells of a 96-well Falcon U-bottom plate. Here, 40,000 efluor 670-stained cancer cells were added to each well. Moreover, 120,000 NK cells were added to each well. Cells were incubated overnight, and in the following morning, cells were spun down, washed, and stained with propidium iodide (PI). A total of 10,000 allophycocyanin (APC)-positive events were collected, and the experiment was performed in triplicate. Supernatant from overnight incubation of cancer cells and NK cells was collected after the first spin prior to washing and PI staining. This supernatant was utilized for LEGENDPlex NK/T Cell Panel Analysis (BioLegend) per protocol. LEGENDPlex values from flow cytometry were back calculated to pg/ml utilizing standard curve interpolation on Prism (see *Statistical Methods* section).

### *In Vitro* Cell Viability Assays

Cells were washed with PBS and stained with PI, which was subsequently analyzed *via* the PE channel on flow cytometry. Alternatively, cells were stained with trypan blue (Sigma) and analyzed *via* Countess (Invitrogen) hemocytometer. Counts were performed in triplicate.

### Flow Cytometry

CD69 data collection and analysis were performed using the Accuri C6 flow cytometer and software (BD Biosciences). Cytoflex (Beckman-Coulter) was utilized for data collection of receptor, cytosolic O-GlcNAc expression, cytotoxicity, viability, and cytokine experiments. Analysis was performed on FlowJo software. The following antibodies (BioLegend) were used with listed conjugations and clones in parentheses: CD3 (FITC, HIT3a), CD56 (APC and FITC, 5.1H11), KIR2DL1 (PerCP/Cy5.5, HP-MA4), KLRG1 (PE, 14C2A07), NKG2A (PE, S19004C), NKp30 (PE, p30-15), NKp44 (PerCP/Cy5.5, p44-8), and NKG2D (PerCP/Cy5.5, 1D11). IgG1 Kappa Isotype (Alexafluor647, P3.6.2.8.1), and O-GlcNAc (Alexafluor 647, RL2) flow antibodies were purchased from Thermofisher Scientific. shOGA and shGFP cells were not examined until at least 7 days after puromycin selection. Intracellular O-GlcNAc levels were examined by flow cytometry every other week and confirmed to still be significantly elevated in shOGA before any other downstream analysis was performed. Intracellular staining was performed using Foxp3 intracellular staining kit (00-5523-00, Thermo Fisher). For the experiment, free GlcNAc (A3256-25G, Sigma-Aldrich) competed with the anti-O-GlcNAc antibody mentioned above for binding. NK cells were isolated and incubated with the antibody for 45 mins with or without 50mM, 250mM, and 500mM GlcNAc, and O-GlcNAcylation detection was assessed *via* flow cytometry.

### Western Blot

Protein was isolated with RIPA buffer (Sigma) with protease and phosphatase single-use inhibitor (78442, Thermo Fisher). Western blots were performed using Bio-Rad mini protean system. Depending on the experiment, 20–35 µg of protein was loaded per well and diluted with 4× Laemmli buffer (Bio-Rad). Beta-actin was used as a loading control (SC-47778, Santa Cruz). O-GlcNAc antibody (ab2739, Abcam) was utilized. Chemiluminescence was performed with systems from Pierce according to protocol and imaged on Bio-Rad Chemidoc XRS+ and analyzed on Quantity One (Bio-Rad). **Figure 1** Western blots were performed in a dark room with X-ray film and SRX-101A developer (Konica Minolta). Antibodies used in **Figure 1** and developed in the dark room are as follows: GADPH (sc47724), GFAT1 (sc377479), GNA1 (sc374519), PGM3 (sc390239), OGT (sc32921) from Santa Cruz, and UAP1 (ab95949) from Abcam. Antibodies used in **Supplementary Figure S2** are as follows: NF-κB (8242T), NFAT1 (5861T), p-AKT at T308 (13038T), pan AKT (4691), ERK/p42/p44 (4695T), p-ERK T202/Y204 (9101S), PLCgamma2 (3872T), STAT5 (25656), and p-STAT5 Y694 (9351S)—all from Cell Signaling Technology normalized to beta-actin from Santa Cruz mentioned above. Western blots were quantified using ImageJ software. Area under the curve was determined and normalized to respective loading control’s [beta actin or Glyceraldehyde-3-Phosphate Dehydrogenase (GAPDH)] area under the curve.

### Statistical Analysis

Statistical analysis was conducted *via* GraphPad Prism software version 9. Statistical tests described in figure legends were used for respective experiments. Asterisks were used to display varying levels of significance from p < 0.05 to p < 0.0001 as described in figure legends. A summary of statistical methods used is as follows: Student’s unpaired T-test and one-way ANOVA with Dunnett’s multiple comparisons to the NK group mean or HEK293T cells that were not transfected. Standard curve creation for LEGENDPlex Analysis was performed utilizing sigmoidal 4PL, X is concentration as interpolation setting. *In vitro* experiments were performed 2–4 times with at least 3 technical replicates per experiment. *In vivo* experiments were performed with the number of mice indicated by (n) per group.

## Results

### Cytokine Activation Increases O-GlcNAcylation in Natural Killer Cells

O-GlcNAcylation is the end product of the HBP, where glucose is converted to UDP-GlcNAc and OGT transfers GlcNAc from UDP-GlcNAc to substrate proteins and OGA removes it (**Figure 1A**). We examined the expression levels of enzymes involved in the HBP pathway before and after stimulation with IL-2 in primary NK cells. As shown in **Figure 1B** and **Supplementary Figure S1A**, OGT, GlcNAc phosphomutase (PGM-3), glutamine fructose-6-phosphate amidotransferase-1 (GFAT-1), UDP-N-acetylglucosamine pyrophosphorylase (UAP-1), and glucosamine-6-phosphate N-acetyltransferase (GNA-1) levels were increased in NK cells activated by IL-2. We also confirmed that the viability of NK cells with and without the cytokine is comparable (**Figure 1C**). To confirm whether IL-2 treatment increases O-GlcNAcylation, we performed Western blotting and found an increased level of global O-GlcNAcylation after 24 h of stimulation of freshly isolated NK cells with IL-2. Similarly, an increase in O-GlcNAcylation was noticed after 48 h of IL-2 or IL-15 treatment of cultured expanded NK cells (**Figure 1D** and **Supplementary Figure S1B**). Activation of NK cells after cytokine stimulation was confirmed by an increase in CD69 expression, a marker of NK cell activation (**Figure 1E**). This shows that an increase in O-GlcNAcylation is associated with cytokine-induced NK cell activation.

OSMI-1 and TG are well-established inhibitors of OGT and OGA, respectively. We measured O-GlcNAc protein levels by Western blotting and found marked reduction of O-GlcNAc levels with OSMI-1 (25 μM) and marked increase of O-GlcNAc levels with TG (50 μM), both after 24 h of incubation (**Figure 1F**, **Supplementary Figure S1C**). Next, we examined the decrease or increase in intracellular O-GlcNAcylation by flow cytometry after incubation of cells with OSMI-1 and TG, respectively, using an anti-O-GlcNAcylated flow cytometry antibody (RL-2) (**Figures 1G, H** **)**. To ensure that this antibody specifically binds only to intracellular O-GlcNAcylated proteins, we tested its binding to surface proteins on NK cells and found no detection of O-GlcNAc signal on the cell surface of NK cells (**Figure 1I**). To further validate this antibody, a competition assay was performed by adding free GlcNAc at increasing concentrations, 50, 250, and 500 mM, for 45 min during the staining with RL2 antibody, and O-GlcNAc levels were detected by flow cytometry. We found a decreased detection of O-GlcNAc even at the lowest tested concentration, 50 mM GlcNAc, which further went down in the presence of 250 and 500 mM free GlcNAc (**Figure 1J**), confirming the specificity of RL-2 antibody in detecting O-GlcNAc by flow cytometry.

### Inhibition of O-GlcNAcylation Results in Altered Expression of Surface Receptors and Cytokines in Primary Natural Killer Cells

**Figure 2:**
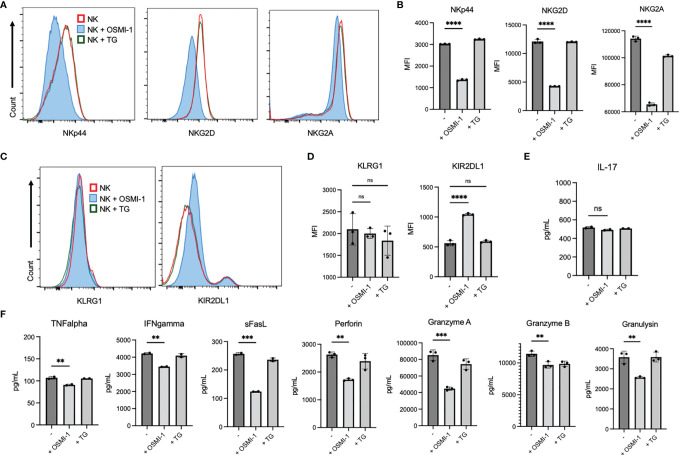
Receptor expression and cytokine secretion are altered in a pattern suggestive of decreased cytotoxicity with OSMI (25 µM) but not TG (50 µM) treatment. **(A–D)** Primary NK cells were treated with OSMI-1 (25 µM) and TG (50 µM) for 24 h, and surface receptor expression was analyzed. Primary cells were gated as CD3 [FITC (Fluorescein isothiocyanate)] negative and CD56 (APC) positive to indicate the NK population. Flowcharts (**A/C**) and graphical representations of MFI **(B, D)** from gated primary NK cells are shown. **(A, B)** Display of receptors that are downregulated. **(C, D)** Display of receptors that display no change or increased surface expression. **(E, F)** Primary NK cells were incubated in OSMI-1 and TG for 36 h, and cytokine expression was analyzed of supernatants at 1:10 and 1:100 dilution depending on cytokine abundance *via* LEGENDplex. Cytokine values calculated off of standard curve and back calculated using their respective dilutions. **(E)** The picograms (pg)/milliliters (ml) of IL-17 expression determined by LEGENDplex and subsequent flow cytometry do not significantly change. **(F)** Cytokines that are significantly decreased in the NK + OSMI-1 (25 µM) treatment group as determined by LEGENDplex and subsequent flow cytometry. Receptor analysis and cytokine analysis was determined *via* one-way ANOVA with Dunnett’s multiple comparisons to the untreated (-) NK group mean. NS indicates no significance. ** indicates significance p <.01, ***p <.001, ****p <.0001. Error bars are standard deviation of the mean. Experiments 2A and 2D were performed three times, and 2E was performed twice. Individual values represent individual cell culture wells. Experiments chosen are representative.

Cell surface receptors expressed on NK cells act as a sensory system that initiates downstream signaling after binding to its respective ligands, which determines the cellular response of NK cells. To determine whether alteration of O-GlcNAcylation in NK cells results in changes in receptor expression, we treated primary NK cells with OSMI-1 or TG for 24 h and measured surface receptor expressions *via* flow cytometry. We show that surface expressions of activating receptors NKp44 and NKG2D and inhibitory receptor NKG2A are significantly downregulated (**Figures 2A, B** **)**, inhibitory receptor KIR2DL1 expression is upregulated, while KLRG1 expression remains unaltered after OSMI-1 treatment (**Figures 2C, D** **)**. HLA-E, ligand of NKG2A receptor, expression on tumor cells was detected, and 2 out of 3 tumor cells showed very low expression (**Supplementary Figure S2A**). Analysis of the cell signaling pathways [phospholipase C-gamma-2 (PLC-γ2), STAT5, extracellular signal-regulated kinase 1/2 (ERK1/2), AKT] and transcription factors [nuclear factor-κb (NF-κb), Nuclear factor of activated T-cells (NFAT)] involved in NK cell function showed some minor alterations after OSMI-1 treatment (**Supplementary Figures S2B–E**) that needs further confirmation using NK cells from multiple donors. Cytokines secreted by NK cells also play a crucial role in antitumor activity. To determine the cytokine secretion levels, primary NK cells were treated as above and culture supernatants were analyzed *via* LEGENDplex (BioLegend). IL-17 levels did not show any changes (**Figure 2E**), while decreased levels of TNF-α, IFN-γ, soluble Fas ligand, perforin, granzyme A, granzyme B, and granulysin were observed after OSMI-1 treatment (**Figure 2F**). Interestingly, TG treatment did not cause significant alterations in receptor or cytokine secretion after 48 h (**Figures 2A–F**).

### Inhibiting O-GlcNAcylation Inhibits Primary Natural Killer Cell Cytotoxicity *In Vitro*

**Figure 3:**
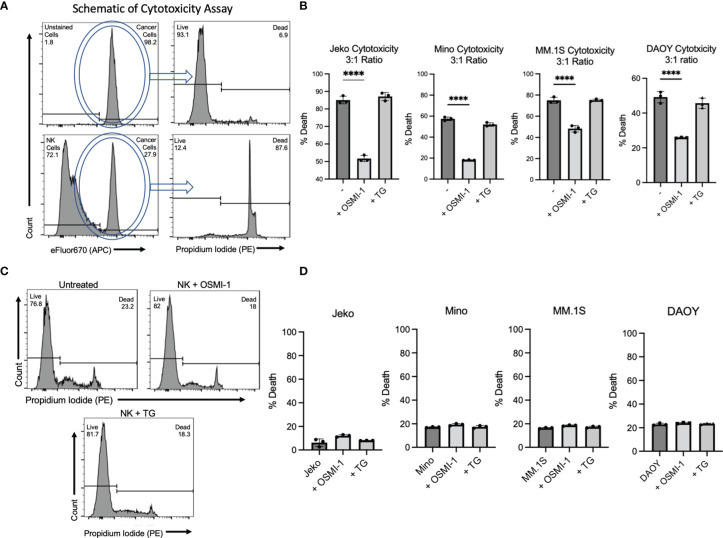
OSMI-1 (25 µM) treatment inhibits primary NK and NK92 cytotoxicity *in vitro*, while TG (50 µM) has no effect on cytotoxicity. Panel **(A)** shows schematic of flow cytometry gating strategy with efluor670-labeled cancer cells visible in APC channel and subsequent gating of PI stain peak. The top two panels show Jeko cells without NK cells present take up high rates of the dye and are largely viable. The bottom two panels show that the APC channel can distinguish between stained Jeko cells and unstained NK cells, and in the presence of NK cells, a far greater number of Jeko cells are dead. Panel **(B)** shows cytotoxicity of primary NK cells pretreated for 24 h with OSMI-1 (25 µM) and TG (50 µM) before being coincubated with Jeko, Mino, and MM.1S cells for 12 h and DAOY for 4 h with OSMI-1 (25 µM) and TG (50 µM) still present. Panel **(C)** shows Jeko, Mino, and MM.1S cell death as a result of OSMI-1 (25 µM) and TG (50 µM) treatment for 24h and DAOY at 12 h. Panel **(D)** shows viability of NK cells after 48 h of culture with OSMI-1 (25 µM) and TG (50 µM). Statistical analysis determined *via* one-way ANOVA with Dunnett’s multiple comparisons to the untreated (-) NK group mean. **** indicates significance of p <.0001. Error bars are standard deviation of the mean. Experiments shown in 1C, as well as those done on Jeko, Mino, and MM.1S cells in 1B and 1D were performed three times. Experiments performed on DAOY cells were done twice. All three individual data points displayed are individual cellular cultures or cocultures of cytotoxicity experiments. Experiments shown are representative of 2 or 3 experiments performed as indicated above.

Given the changes in surface receptor profile and cytokine secretion, we investigated whether NK cell’s antitumor cytotoxicity was affected following OSMI-1 treatment. Primary NK cells were incubated with OSMI-1 or TG for 24 h and then coincubated at a 3:1 ratio with efluor-670-stained mantle cell lymphoma (MCL) cell lines Jeko and Mino or multiple myeloma (MM) cell line MM.1S or medulloblastoma cell line DAOY. After 12 h of coincubation for hematologic malignancies and 4 h of coincubation for the solid tumor DAOY, we analyzed the percent tumor cell death. An example of the cytotoxicity gating on APC (efluor-670)-stained cancer cells and their PI uptake is shown in **Figure 3A**. This experiment showed substantial and statistically significant decreases in cytotoxicity (30%–65%) by NK cells after OSMI-1 treatment compared to control NK cells. No significant alterations in cytotoxicity were seen in NK cells post TG treatment (**Figure 3B**). We checked the viability of NK cells themselves after 36 h with OSMI-1 or TG treatment and found no major differences in cell viability after OSMI-1/TG treatments (**Figure 3C**). Finally, we also examined whether OSMI-1 and TG have any direct effects on cancer cells. No major cell death was noticed after OSMI-1 or TG treatment for 12 h (**Figure 3D**).

### NK92 Cells Show Similar Inhibition in Cytotoxicity After OSMI-1 Treatment

**Figure 4:**
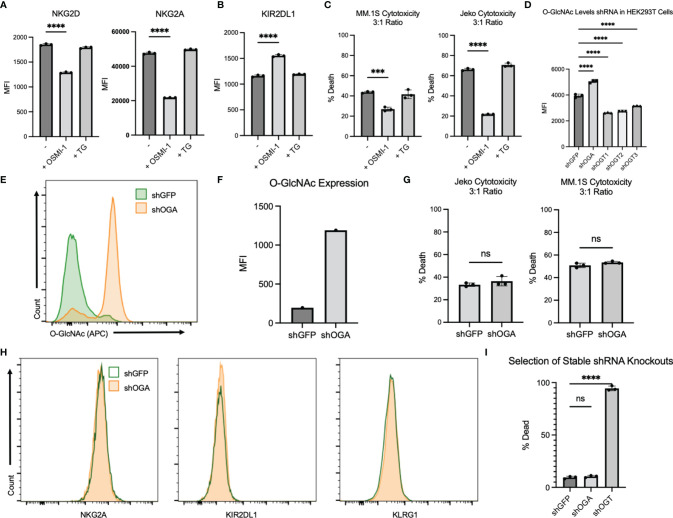
NK92 cells display a similar phenotype to that of primary NK cells after OSMI-1 (25 µM) and TG (50 µM) treatment. Stable shOGA generation in NK92s displays similar results to TG-treated NK92s. **(A)** Two prominent receptors for cytotoxic function after 24 h of treatment with OSMI-1 (25 µM) or TG (50 µM) displayed as MFI representations of flow analysis. **(B)** KIR2DL1 displayed as MFI representation of flow cytometry analysis from the same experimental design as **(A)**. **(C, G)** NK92 **(C)** and shGFP/shOGA **(G)** cytotoxicity displayed as % Death (PI-positive cells detected on PE channel) on flow cytometry using the same gating strategy and design as **Figure 3A** against Jeko and MM.1S cells at 3:1 NK-to-target ratio. **(D)** Intracellular O-GlcNAc levels shown on flow cytometry 30 h post transfection in HEK293T cells. **(E)** Displays intracellular O-GlcNAc expression *via* flow cytometry of shOGA stable knockdown vs. shGFP control. **(F)** Graphical representation of flow cytometry shown in panel **(E)**. **(H)** Prominent receptors of NK cell cytotoxicity displayed in shGFP vs. shOGA cells 10 days after puromycin selection was completed. **(I)** Displays data showing cell death based on PI staining on flow cytometry for shGFP, shOGA, and shOGT after transduction 6 days after puromycin (2 µg/ml) selection has begun. Statistical analysis for receptors and NK92 cytotoxicity determined *via* one-way ANOVA with Dunnett’s multiple comparisons to the NK group mean. Significance for shGFP vs. shOGA cytotoxicity determined by unpaired Student’s T-test. NS indicates no significance. *** indicates significance of p <.001 and **** indicates p <.0001. Error bars are standard deviation of the mean. Experiments 4A–C were performed three times. Experiments 4D–I were performed twice. The three individual values displayed are cell cultures within one experiment, and experiments displayed are representative of all experimental results of replicates.

It is challenging to generate stable cells after viral transductions of primary NK cells (26). Hence, we chose the NK cell line NK.92 to do genetic modifications. First, we wanted to confirm if OSMI-1 and TG treatments had similar effects in NK.92 cells as seen in primary NK cells. We examined 3 receptors, NKG2D, NKG2A, and KIR2DL1 and found that the expression of NKG2D and NKG2A decreased (**Figure 4A**) while KIR2DL1 increased (**Figure 4B**) after OSMI-1 treatment, similar to primary NK cells. We found no significant changes in expression of these receptors after TG treatment (**Figures 4A, B** **)**. In addition to this, a significant reduction in NK.92 cell cytotoxicity after OSMI-1 treatment was observed after coincubation with two different cancer cells Jeko-1 and MM1.S (**Figure 4C**).

We validated one shOGA and three shOGT plasmids by transducing HEK293T cells. shOGA-transduced cells showed a significant increase and shOGT-transduced cells showed a significant decrease in O-GlcNAcylation as measured by flow cytometry (**Figure 4D**). We then created a stable NK.92 cell line expressing shOGA or shGFP as a control. We found a substantial increase in the O-GlcNAcylation levels in shOGA cells compared to shGFP control cells (**Figures 4E, F**; **Supplementary Figures S3A–C**). We used the shOGA cell line to validate the data obtained after TG treatment in NK.92 cells. We determined whether shOGA transduction in NK.92 cells resulted in alterations in its cytotoxic function. No alterations in cytotoxicity were seen between the shGFP and shOGA stable cell lines when cocultured with Jeko or MM.1S cancer cells (**Figure 4G**). Next, we checked the surface receptor analysis by flow cytometry. We did not see any major changes in NKG2A, KIR2DL1, and KLRG1 receptor expression (**Figure 4H**). These shOGA-transduced cells behaved similarly to the TG-treated NK cells and confirm that the increase in O-GlcNAcylation in NK cells results in no major changes in cytotoxic activity by using two approaches.

Our attempts to create shOGT stable knockdown were unsuccessful, despite successful generation of shGFP- and shOGA-transduced NK.92 cell lines. shOGT-transduced NK.92 cells died after puromycin selection, while shGFP and shOGA cells were viable as evident from PI staining shown in **Figure 4I** and **Supplementary Figure S3C**. This implies that O-GlcNAcylation is required for NK92 cell viability.

### OSMI-1 Inhibits Primary Natural Killer Cell Cytotoxicity *In Vivo*

**Figure 5:**
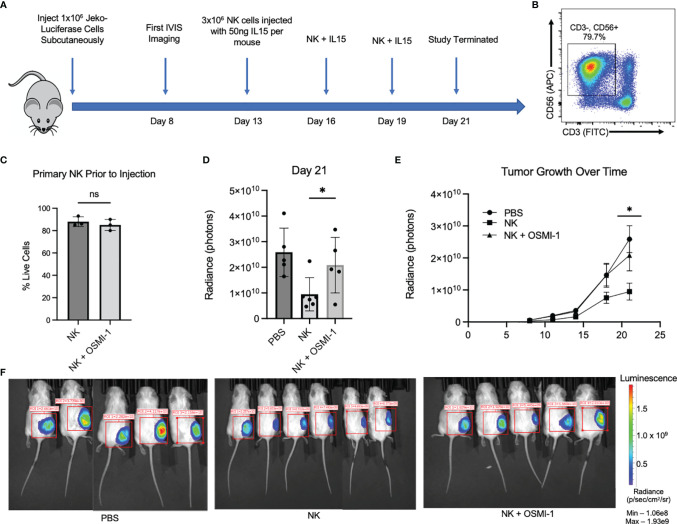
OSMI-1 pretreatment of primary NK cells inhibits cytotoxicity *in vivo.* Mice were inoculated with 1 × 10^7^ Jeko-luciferase cells subcutaneously. Beginning at Day 11, mice were injected with PBS (sham) or 3 million primary NK cells with or without 36 h of incubation with OSMI-1 (25 µM) every 3 days with *In Vivo* Imaging System (IVIS) measurements being taken every 3–4 days. IL-15 was added to cell suspension at 50 ng per mouse just prior to injection. **(A)** Schematic representation of *in vivo* experimental design. Panel **(B)** shows primary NK cell population at time of injection defined as CD3-/CD56+. **(C)** Primary NK live/dead staining 1:1 with trypan blue *via* Countess after 36-h incubation with OSMI (25 µM) taken just prior to injection. **(D)** Radiance (photon) expression of IVIS imaging of individual mice at Day 21 normalized to same scale. **(E)** Line graph of average increase per treatment group in photons per constant ROI as measured by IVIS with error bars displaying standard error of the mean. Panel **F** shows luciferase IVIS images for mice at Day 21. PBS and OSMI-1 groups had n = 5, and NK-only group had n = 6. Images normalized to same IVIS spectrum sensitivity range. NS indicates no significance. * indicates significance of p <.05 as determined by Student’s unpaired T-test between untreated NK and NK + OSMI-1 group. Experiments in panels **(B, C)** were performed once at each indicated time point. **Figure 1C** has 3 individual data points from samples taken from the cellular population prior to injection. 1B is representative across the 3 time points of injections.

We used a mantle cell lymphoma, Jeko-1 cell, xenograft model to study *in vivo* cytotoxicity of NK cells pretreated with OSMI-1 compared to control NK cells. NSG mice were injected with Jeko cells expressing luciferase subcutaneously and NK cells (control NK or NK cells pretreated with OSMI-1) were injected intratumorally on days 13, 16, and 19, and mice were sacrificed on day 21. A schematic of the experiment is shown in **Figure 5A**. Primary NK cells (CD3^-^ CD56^+^) (**Figure 5B**) were tested to ensure good viability 15 min before injection and that viabilities were not significantly different in control and OSMI-1-treated groups after 36 h of treatment (**Figure 5C**). Luciferase image quantification at Day 21 is shown as radiance (**Figure 5D**). Tumor growth evaluated by radiance is charted through Day 21, and imaging was done twice weekly. This imaging shows that NK cell-treated mice had overall reduced tumor burden while tumors progressed in mice injected with OSMI-1-treated NK cells similar to PBS-injected mice (**Figure 5E**). Luciferase mouse imaging at Day 21 is shown (**Figure 5F**). Overall, the *in vivo* data validated the *in vitro* data that OSMI-1-mediated suppression of O-GlcNAcylation inhibits NK cell cytotoxicity.

## Discussion

Increased O-GlcNAcylation is found in several types of cancers (27, 28). Whether or not O-GlcNAcylation plays a role in the anticancer cytotoxic function of immune cells and how much it contributes to cancer progression still remain to be detailed. We studied the role of O-GlcNAcylation in NK cells and found that enzymes involved in the HBP pathway and O-GlcNAcylation are upregulated in NK cells after cytokine stimulation. Inhibition of O-GlcNAcylation following OSMI-1 treatment resulted in a decrease in O-GlcNAcylation and inhibition of NK cell cytotoxic function. This phenotype can be attributed partially to the downregulation of NK cell-activating receptors NKG2D and NKp44 as well as upregulation of KIR2DL1 inhibitory receptors on NK cells. KLRG1 receptor expression was not altered after OSMI-1 treatment, suggesting that only selected receptors are under the regulation by O-GlcNAcylation in NK cells. It is interesting to note that the increase in O-GlcNAcylation after TG treatment did not show any effect on the expression of receptors, cytokines, and cytotoxic mediators in NK cells or alter the NK cell cytotoxic function. In order to account for NK cell and tumor cell heterogeneity, we performed this experiment using four different cancer cell lines and human NK cells from three different donors. We further confirmed this observation by transducing shOGA in NK cells. As expected, inhibiting OGA increased O-GlcNAcylation in NK cells and did not affect cytotoxicity of NK cells.

Both phosphorylation and O-GlcNAcylation happen in serine and threonine residues of proteins (29), and hence, inhibiting O-GlcNAcylation might result in increased phosphorylation of proteins and increased O-GlcNAcylation might shift the equilibrium away from phosphorylation, accounting for the observed changes in NK cell cytotoxic function after OSMI-1 treatment. In line with this, it has been shown that a disruption in O-GlcNAcylation equilibrium poses serious risks in disease conditions like Alzheimer’s disease, where O-GlcNAcylation changes alter phosphorylation levels of Tau protein, which is involved in the pathogenesis of the disease (30, 31). More studies are needed to address the correlation between O-GlcNAcylation, phosphorylation, and NK cell function in physiological and pathological conditions including Alzheimer’s disease, cancer, and diabetes where significant alterations in O-GlcNAcylation are observed (32–38). Though NK cell dysfunction in diabetes patients is reported, more studies are needed to test whether any connection exists between O-GlcNAcylation status of NK cells and decreased cytotoxic function of NK cells observed in these patients.

Inhibition of activating receptors, cytokines, and cytotoxic mediators after inhibition of O-GlcNAcylation in NK cells might be resulting from changes in O-GlcNAcylation of various transcription factors. NF-κB and NFAT are transcription factors known to be O-GlcNAcylated (39–41) and reported to be playing important roles in immune cell activation and function (42–52). Multiple NF-κB members are reported to be involved in NK cell activation, cytokine production, and cytotoxic function (42–45). We recently reported the role of NF-κB c-Rel in regulating NK cell cytotoxic function (43). Inhibiting c-Rel in NK cells led to reduced expression of perforin and granzyme, resulting in decreased cytotoxicity. NF-κB c-Rel is known to be O-GlcNAcylated on its Ser350 residue and is involved in the activation of genes in helper T-cell activation (46) and suppression of FOXP3 in T regulatory cells (47). Previous reports have shown that OGT knockdown inhibited both NF-κB and NFAT activation in T cells (48), along with decreased production of IL-2 post T-cell Receptor (TCR) activation. A similar effect was also observed in OGT-knockout mouse T cells, which failed to proliferate after IL-2 stimulation (49, 50). NFAT has been shown to translocate to the nucleus from cytosol following NK cell stimulation (51). Whether O-GlcNAcylation of NFAT plays a role in NK-cell function remains to be determined. Thus, further studies are needed to address the involvement of NF-κB and NFAT transcription factors and its O-GlcNAcylated forms in the NK cell cytotoxic function.

Since NK cells are a minor population of immune cells, we used flow cytometry to detect overall O-GlcNAcylation in NK cells and validated this by Western blotting. We confirmed that the signal observed in flow cytometry are indeed from intracellular O-GlcNAcylation by confirming that no cell surface staining was detected using the anti-O-GlcNAcylated antibody used for flow cytometry. As expected, the O-GlcNAcylation signal decreased after OSMI-1 treatment and increased after TG treatment and in shOGA-transduced cells, validating the specificity of the antibody. This flow cytometry method will be a useful tool for future O-GlcNAcylation studies on rare cell populations like NK cells.

Due to poor transfection efficacy in NK cells, siRNA OGT transfection or nucleofection was also not an option to achieve OGT knockdown in these cells. However, shOGA- and shGFP-transduced NK92 cells were viable. For *in vivo* experiment, we did not inject OSMI-1 directly into mice, as it will affect O-GlcNAcylation in other cell types. Our *in vivo* experiments using NK cells pretreated with OSMI-1 confirms our *in vitro* findings that inhibition of O-GlcNAcylation in NK cells is associated with inhibition of NK cell cytotoxicity.

In our experiments, we found that the shOGT-transduced NK cells were not viable. Previous studies also have shown that OGT expression is essential for cell viability (52–54). This points to a possibility that NK cells require a certain degree of O-GlcNAcylation for their survival. O-GlcNAc homeostasis or an optimal level of O-GlcNAcylation is required for proper maintenance of signaling pathways in cells (30, 31). A minimum level of O-GlcNAcylation in certain proteins is needed for proper cell functioning, and these levels might be maintained by the expression and activity levels of OGT and OGA enzymes. Our data point to the possibility that there is an optimal level of O-GlcNAcylation critical for NK cell cytotoxic function, and if those levels are inhibited, it leads to a decrease in cytotoxic function, while increases in the O-GlcNAcylation levels of proteins do not necessarily shift the balance the other way. These results also suggest that cancer therapeutics that focus on targeting elevated O-GlcNAc levels in cancer cells (55) should weigh the risks associated with potential decreases in NK cell (and other immune cell) effector function.

## Data Availability Statement

The original contributions presented in the study are included in the article/**Supplementary Material**. Further inquiries can be directed to the corresponding author.

## Ethics Statement

The studies involving de-identified human blood samples were reviewed and approved as IRB exempt by University Hospitals IRB. Written informed consent for participation was not required for this study in accordance with the national legislation and the institutional requirements. The animal study was reviewed and approved by Institution’s Animal Care and Use Committee, Case Western Reserve University.

## Author Contributions

DF performed the experiments, analyzed data and wrote the manuscript. PR contributed to study design, discussions, troubleshooting as well as writing and editing the manuscript. DW performed clonings, viral transductions and NK cell expansions, AA performed some of the experiments in **Figure 1**. RP conceived the study, oversaw the study, designed experiments, guided research personnel, performed data analysis and wrote the manuscript. All authors contributed to the article and approved the submitted version.

## Funding

RP was supported by St. Baldrick's scholar award and NIH/NCI R21 CA246194. P.R. was supported by NIH/NIAID grants R01Alll6730, R21All44264 and NIH/NCI grant R21 CA246194. This research was also supported by the Hemopoietic Biorepository and Cellular therapy core facilities, Shared Resources of the Case Comprehensive Cancer Center (P30CA043703). DF was supported by NIH T32GM007250-44 and T32GM007250-45. DW was supported by T32GM007250-43.

## Conflict of Interest

RP is a consultant for Luminary Therapeutics.

The remaining authors declare that the research was conducted in the absence of any commercial or financial relationships that could be construed as a potential conflict of interest.

## Publisher’s Note

All claims expressed in this article are solely those of the authors and do not necessarily represent those of their affiliated organizations, or those of the publisher, the editors and the reviewers. Any product that may be evaluated in this article, or claim that may be made by its manufacturer, is not guaranteed or endorsed by the publisher.

## Supplementary Material

Supplementary Figure 1 ImageJ Quantifications of western blots in Figure 1. **(A)** – Graphical representation of western blot images from Figure 1B. Analysis/normalization was performed as described in Figure 1. ImageJ was utilized to determine area under curve for each band, and each band was normalized to the area under the curve of its lane’s loading control. **(B)** – Display of western blot images from Figure 1D. **(C)** – Graphical representation of western blot shown in in Figure 1F.

Supplementary Figure 2 HLA-E expression in cancer cells and protein expression of transcription factors and prominant pathways in primary NK cells. **(A)** – HLA-E surface expression detected on three hematologic cancer cell lines detected by flow cytometry and displayed as MFI. **(B)** – Protein expression shown *via* western blot of primary NK cells that received no treatment (NT), OSMI (25uM), or TG (50uM) for 24 hours prior to protein harvest. **(C)** – Graphical representation of B showing quantitation performed in ImageJ by taking the area-under-the-curve. All experiments were performed twice. Experiments displayed are representative of experiments performed.

Supplementary Figure 3 Western blot of shOGA compared to shGFP and Flow Imaging of PI stains in shRNA vectors. **(A)** Displays western blot of protein O-glcnacylation levels in shGFP and shOGA cells with beta-actin loading control. **(B)** Graphical representation of the western blot using ImageJ quantification software by taking the area-under-the-curve. Normalization was performed by dividing O-GlcNAc levels by Beta-Actin levels of each respective lane. **(C)** Propidium Iodide staining representative image taken from one individual data point of flow cytometry on puromycin selected NK92 cells.
