# Supplementary figures and images for: Inhibition of O-GlcNAcylation Decreases the Cytotoxic Function of Natural Killer Cells
Source: Front Immunol. 2022 Apr 11;13:841299. doi: 10.3389/fimmu.2022.841299 (PMC9036377; doi:10.3389/fimmu.2022.841299)

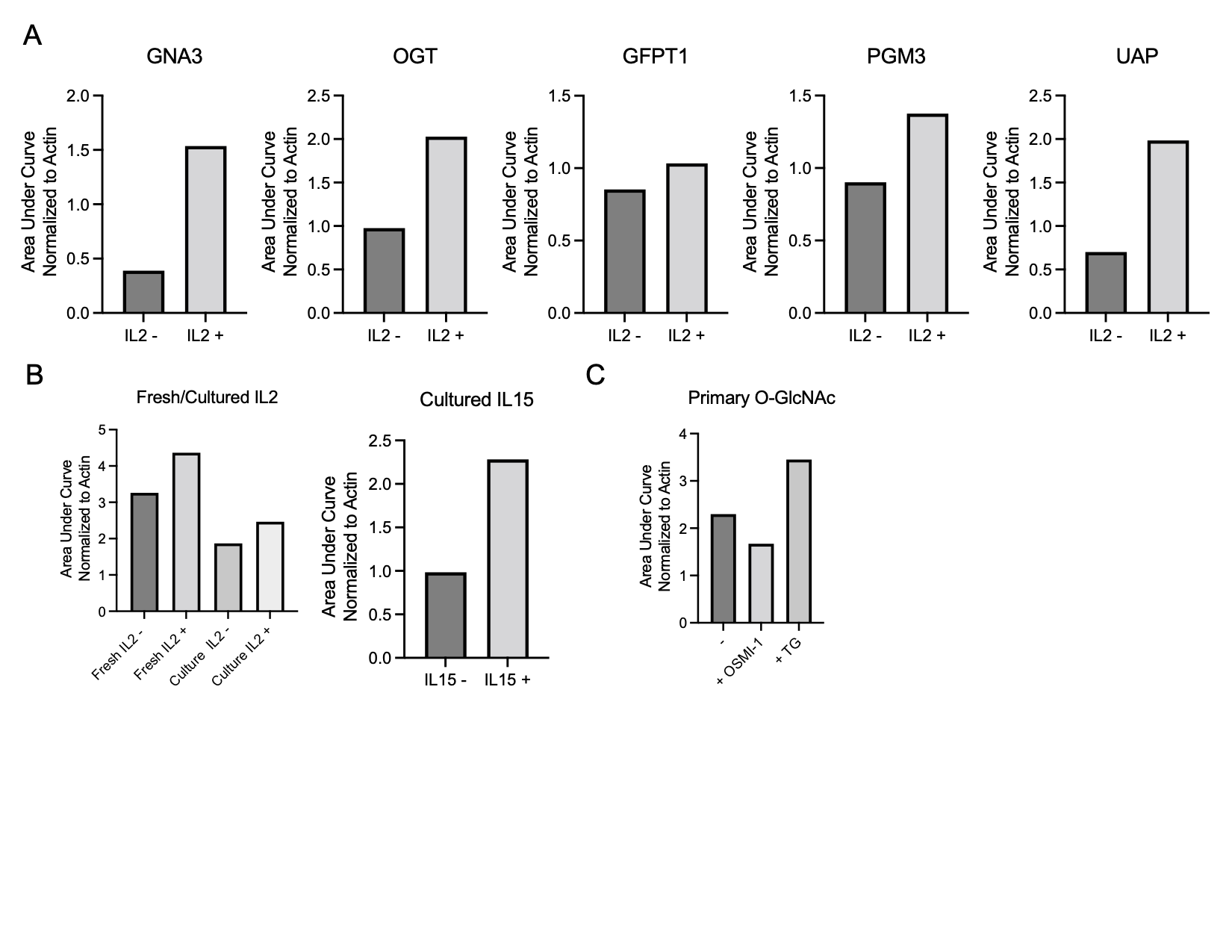

Supplement: Supplementary Figure 1 — ImageJ Quantifications of western blots in Figure 1. (A) – Graphical representation of western blot images from Figure 1B. Analysis/normalization was performed as described in Figure 1. ImageJ was utilized to determine area under curve for each band, and each band was normalized to the area under the curve of its lane’s loading control. (B) – Display of western blot images from Figure 1D. (C) – Graphical representation of western blot shown in in Figure 1F. [file Image_1.tiff]

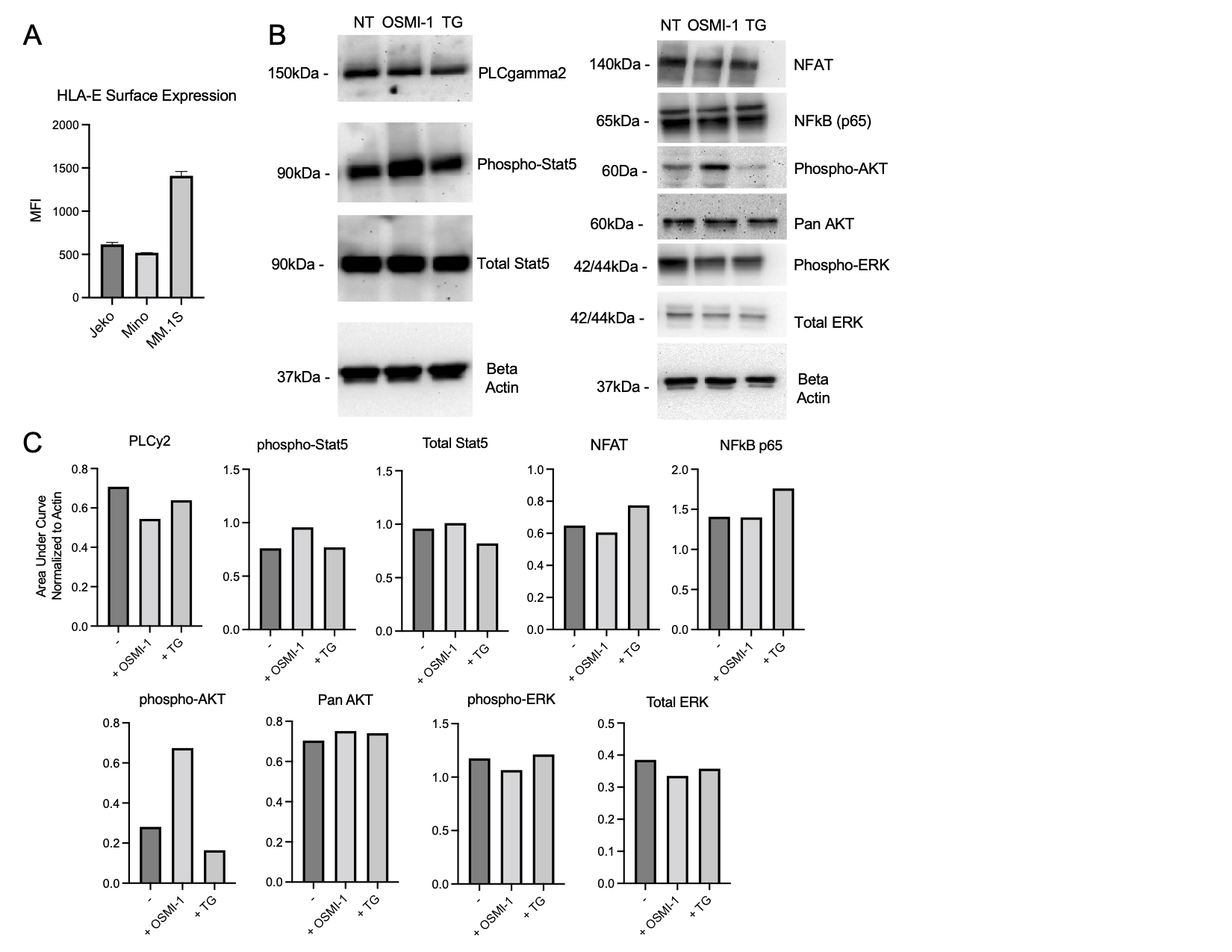

Supplement: Supplementary Figure 2 — HLA-E expression in cancer cells and protein expression of transcription factors and prominant pathways in primary NK cells. (A) – HLA-E surface expression detected on three hematologic cancer cell lines detected by flow cytometry and displayed as MFI. (B) – Protein expression shown via western blot of primary NK cells that received no treatment (NT), OSMI (25uM), or TG (50uM) for 24 hours prior to protein harvest. (C) – Graphical representation of B showing quantitation performed in ImageJ by taking the area-under-the-curve. All experiments were performed twice. Experiments displayed are representative of experiments performed. [file Image_2.tiff]

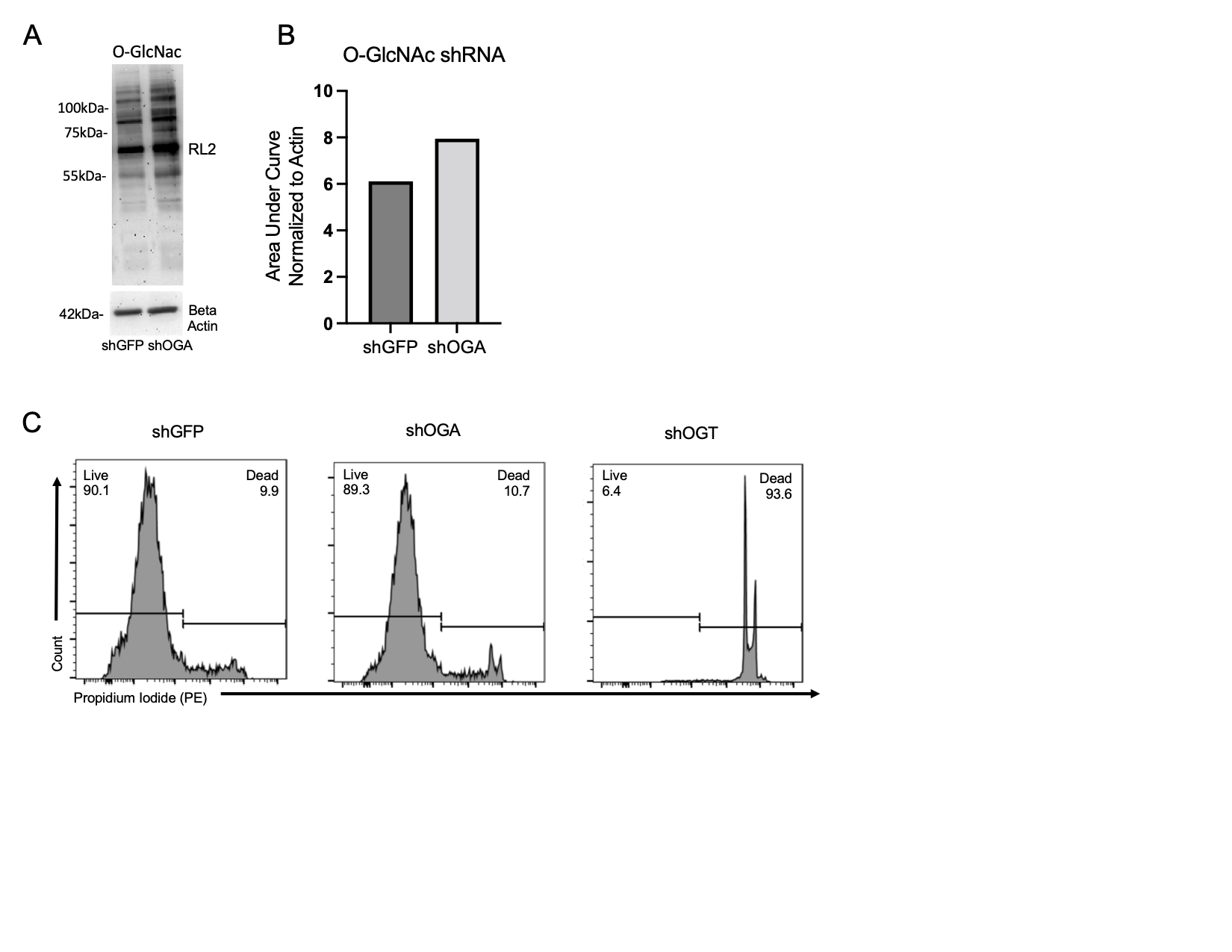

Supplement: Supplementary Figure 3 — Western blot of shOGA compared to shGFP and Flow Imaging of PI stains in shRNA vectors. (A) Displays western blot of protein O-glcnacylation levels in shGFP and shOGA cells with beta-actin loading control. (B) Graphical representation of the western blot using ImageJ quantification software by taking the area-under-the-curve. Normalization was performed by dividing O-GlcNAc levels by Beta-Actin levels of each respective lane. (C) Propidium Iodide staining representative image taken from one individual data point of flow cytometry on puromycin selected NK92 cells. [file Image_3.tiff]
